# Impact of Flipped Classroom Instruction on Brain-Mediated Motor Skill Performance in University Students: A Systematic Review and Meta-Analysis

**DOI:** 10.3390/brainsci15050501

**Published:** 2025-05-14

**Authors:** Kerui Liu, Zikang Hao, Jiping Chen, Qingxu Wu, Wei Jin, Yang Pan, Xianliang Zhang

**Affiliations:** School of Physical Education, Shandong University, Jinan 250061, China

**Keywords:** sports performance, flipped classroom model, university education, physical education, meta-analysis

## Abstract

Objective: This systematic review and meta-analysis evaluates how the flipped classroom model—considered as a neurocognitive training environment—affects cognitive–motor integration and brain-mediated motor skill performance in university students, providing scientific evidence for optimizing higher-education physical education pedagogy (a course related to physical literacy and the cultivation of physical and mental health, rather than a training program for professional physical education teachers). Methods: In order to compare the effects of flipped classroom and traditional teaching on the motor skill performance of university students, this study conducted a systematic review and meta-analysis according to PRISMA rules, whereby studies were screened according to specific inclusion criteria and data were extracted, assessed for quality, and then meta-analyzed to assess the effectiveness of the flipped classroom model in improving motor skill performance. Results: A total of 12 original randomized controlled trials (RCTs) were included in the study. The meta-analysis results indicated that the flipped classroom model significantly outperformed traditional teaching methods in improving university students’ motor skill scores (standardized mean difference (SMD) = 1.22, 95% CI = 0.64–1.79, *p* < 0.0001). Subgroup analysis showed significant effects in both general major students and sports science major students, with no significant difference between studies conducted in China and those conducted in non-China regions. Conclusions: The flipped classroom model demonstrates significant advantages over traditional PE teaching methods in improving motor skill performance. It enhances students’ skill acquisition and classroom engagement, showing promising potential for future implementation in university PE programs. Further research should explore the model’s applicability across different sports and student populations, as well as its long-term impact on skill retention and postgraduation sports participation.

## 1. Introduction

Physical education (PE) at the university level serves a distinct role in student development, differing from K–12 physical education. According to the developmental-motor model proposed by David Gallahue [1] and Haubenstricker and Seefeldt [2], basic motor skills are acquired primarily in early childhood, usually between the ages of 2 and 7. These skills are the foundation layer for more complex motor abilities. During adolescence, individuals enter a specialized motor phase where these skills are refined and applied in increasingly complex and game-specific environments. In higher education, physical education may be delivered as a general curriculum course for all students or as specialized training for future physical education teachers rather than a mere extension of school athletics [3]. Internationally, the concept of “physical education” in universities is framed by diverse philosophies. For example, in some Latin American contexts (e.g., Cuba and Mexico), the term “physical culture” is used to denote comprehensive programs in sport and physical education, emphasizing broad-based, multisport knowledge and cultural integration [4]. In Germany and Northern Europe, the philosophy of Bildung views physical education as a vehicle for holistic personal development—focusing on the student’s self-formation and the integrated growth of cognitive, physical, emotional, and social capacities [5]. These global perspectives underscore that higher-education physical education pedagogy is grounded in more expansive educational and philosophical objectives than basic skill instruction alone. Accordingly, university physical education serves not just as skills training but as an academic endeavor tied to physical literacy and the cultivation of both mind and body. Therefore, at the university level, physical education should be concerned not only with the refinement of motor patterns but with their application in strategic, athletic, and cognitively demanding environments that reflect the highest levels of motor skill development.

However, traditional teaching approaches to PE in universities often adopt a teacher-centered model characterized by lecture-based instruction and repetitive practice. This method tends to overlook students’ individual needs and their capacity for active participation [6]. Consequently, this approach often leads to low levels of engagement and interest among students, ultimately hindering the development of their motor skills and the overall effectiveness of the teaching process [7]. Bessa et al. [8] in their wide-ranging review mention a new teaching model, which puts more emphasis on student agency, especially for raising awareness and interest. While traditional lecture-based instruction has historically dominated educational practice, growing evidence highlights its constrained efficacy in contemporary pedagogical contexts [9]. Characterized by teacher-centered knowledge transmission, this model typically relegates students to passive recipients of prepackaged information, with cognitive engagement primarily limited to in-class note-taking and postlecture memorization tasks [8]. Such structural rigidity proves particularly problematic in skill-acquisition domains, where delayed feedback mechanisms—often spanning 24–48 h between classroom demonstration and homework evaluation—impede motor skill consolidation through interrupted error-correction cycles [10].

With the rapid advancement of information technology and the ongoing evolution of educational paradigms, reforming traditional PE methods to stimulate student interest has become a focal point for educators [11].

The flipped classroom, as an innovative teaching model, has garnered widespread attention and application in global educational contexts in recent years [12]. This approach fundamentally reverses the traditional teaching structure, requiring students to acquire foundational knowledge independently before class through resources such as instructional videos and textbooks. Classroom sessions are then dedicated to discussions, practical exercises, and teacher–student interactions, which reinforce knowledge comprehension and application. By placing students at the center of the learning process, the flipped classroom encourages active participation and cultivates both self-directed learning abilities and creative thinking [13]. Although some teachers have adopted a similar teaching style in their classes, giving the initiative or the subjectivity of the class to students, traditional teaching methods are still in widespread operation. Similarly, the flipped classroom teaching style mentioned here does not completely abandon long-standing traditional teaching methods but rather requires innovation and reform. Under the condition that no fundamental problems occur in a class, it reverses the main characters of the class. It is an empowerment or innovation rather than a subversion or abandonment.

Incorporating the flipped classroom model into the process of teaching year-round PE at the university level may have both unique theoretical and practical advantages. First, the inherently practical and interactive nature of PE aligns well with the flipped classroom structure, allowing for optimal use of classroom time for skill development and hands-on practice, thereby improving teaching efficiency [14]. Second, preclass independent study enables students to grasp the theoretical underpinnings of motor skills in advance, facilitating targeted and problem-focused participation during practice sessions [15]. Additionally, the use of multimedia resources, such as instructional videos and detailed demonstrations, enhances students’ engagement and accommodates diverse learning needs across various proficiency levels [16,17].

From the perspective of brain science, in any complex motor skill or physical tasks—such as dribbling in football, completing gymnastic movements, etc.—the brain must combine thinking with movement. Learners need to plan, monitor, and adjust their actions based on rules, strategies, or environmental feedback. These “brain-mediated” motor skills, wherein neural processes such as perception, anticipation, and feedback control largely govern motor output, are crucial for success in the physical education environment. The progress of motor learning research emphasizes that the acquisition of motor skills does not rely purely muscle activity but is supported by significant neural adaptation and plasticity.

Despite the promising prospects of flipped classrooms in university PE, existing research findings remain inconsistent. While some studies have reported significant improvements in students’ motor skill performance, learning motivation, and satisfaction [18], others have highlighted challenges, such as students’ insufficient self-regulated learning abilities and limited access to instructional resources, which may lead to suboptimal outcomes [19]. These discrepancies may be attributed to variations in sample sizes, teaching content, and implementation processes across studies.

As mentioned earlier, there is some debate about the practical effects of flipped classrooms, especially on motor skills. Although there have been some relevant meta-analyses investigating the differences between flipped classrooms and traditional teaching styles, they have focused more on outcomes such as student motivation and self-efficacy [20]. No studies have systematically examined this aspect of motor skills. Meta-analysis, as a statistical approach, integrates the results of multiple independent studies, enhancing the reliability and generalizability of the findings. By employing meta-analysis, this study aims to explore the differences in outcomes between flipped classroom and traditional teaching models in improving university students’ motor skill performance, providing scientific evidence and practical insights to inform the reform of PE in higher education.

Therefore, this study aimed to systematically search and screen randomized controlled trials (RCTs) that compared flipped classrooms and traditional PE teaching models, which are the recognized “gold standard” when exploring the effects of interventions. By employing meta-analysis, this research quantitatively assessed the impact of flipped classrooms on students’ motor skill performance. The findings are aimed at offering valuable references for educators and policymakers, ultimately driving innovation and development in university PE.

## 2. Materials and Methods

This study was conducted in accordance with the PRISMA guidelines for systematic reviews [21,22]. This study has been registered on the PROSPERO website (registration number: CRD42024621383).

Throughout the methodology, our primary use of the approach was determined based on PICOS principles. Participants: We included studies involving college students (in higher education) enrolled in regular physical education classes or physical activity courses. This included both students enrolled in regular college physical activity courses and students enrolled in physical education teacher education courses, but did not include studies of K–12 students or nonstudent adult populations. We defined ‘higher education physical education’ as an organized program of university courses (compulsory or elective) that focuses on the teaching of physical activity, sport, or kinesiology. Intervention: The intervention we focused on was a flipped classroom approach applied to the teaching of physical activity or motor skills. We incorporated any instructional strategy described as a ‘flipped classroom’ or ‘inverted learning’, which typically included self-study of material prior to class (e.g., online lectures, videos, and readings) and use of class time for active learning (e.g., physical practice, small group practice, or problem-solving activities). Comparison: Comparison groups or conditions had to involve either traditional instruction or another baseline pedagogy (e.g., a traditional physical education class format in which instruction and practice took place in the classroom without flipped content). Studies comparing two different flipped classroom implementations without a traditional control group, as well as studies lacking any comparative elements, were excluded. Outcomes: We focused on motor skill learning or performance outcomes (technical proficiency, skill test scores, etc.). Study design: We included controlled trials comparing flipped classroom instruction with a control group in university physical education. Randomized and nonrandomized studies were eligible given the pedagogical nature of the intervention (true randomization in a classroom setting is sometimes challenging).

### 2.1. Inclusion and Exclusion Criteria for the Study

Inclusion criteria were as follows. (1) Study Design: Randomized controlled trials (RCTs) exploring the impact of traditional teaching versus flipped classroom on university students’ motor skill performance. (2) Study Participants: University students currently enrolled in higher education institutions. (3) Intervention Measures: The experimental group used only the flipped classroom PE teaching model, while the control group used only the traditional PE teaching model. Studies with an intervention time of at least >12 weeks. (4) Outcome Measures: Motor skill performance. The study set up flipped classroom teaching as an experimental group and the traditional teaching mode as a control group. Subgroups were set up according to the students’ majors (nonexercise science majors and exercise science majors) to observe the possible influence of prior athletic training experience on the presence of outcomes and according to the region from which the studies originated (non-Chinese and Chinese regions) because there was a possibility of bias in the merger due to the fact that most of the studies were from the Chinese region.

Exclusion criteria were as follows: (1) studies for which full texts and experimental data could not be obtained, as well as duplicate publications; (2) studies not in Chinese or English; (3) studies where significant differences existed in baseline values between the two groups (*p* < 0.05).

### 2.2. Literature Search Strategy

Computer-assisted searches were conducted in PubMed, EMBase, the Cochrane Library, Web of Science, the Chinese Biomedical Literature Database (CBM), the China National Knowledge Infrastructure (CNKI), the VIP Network, and the Wanfang Data Knowledge Service Platform to collect studies on the application of the flipped classroom PE teaching model to improve university students’ motor skill performance. The search period for all databases was from the inception of the database to November 2024. Additionally, references cited in relevant systematic reviews and gray literature were traced, and related journals were manually searched to supplement the literature and ensure comprehensiveness [23].

For example, in PubMed, we used a combination of MeSH terms and free-text terms to capture the concepts of physical education, flipped classroom, cognition, and motor skills. A sample PubMed search string was: (“Physical Education and Training”[MeSH] OR “physical education” OR “physical training” OR “sport”) AND (“Flipped Classroom” OR “flipped learning” OR “inverted classroom” OR “blended learning”) AND (“Motor Skills”[MeSH] OR “motor skill” OR “motor performance” OR “skill acquisition”) AND (cognition OR cognitive OR brain OR “executive function”). Similar search logic, with appropriate syntax adjustments and subject headings, was applied to other databases.

### 2.3. Literature Screening

**Figure 1:**
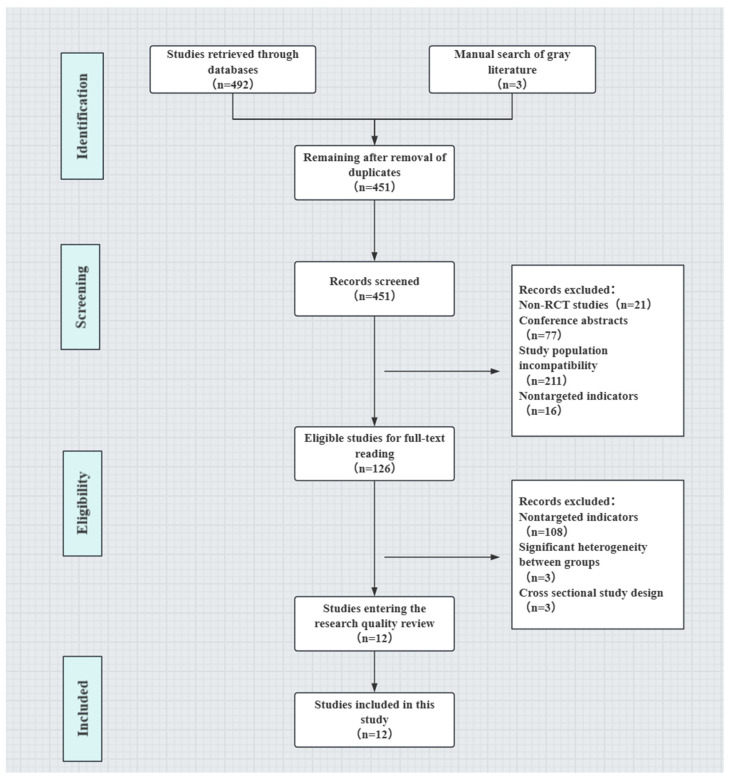
Screening process for this study.

Screening of the literature and data extraction involved importing the retrieved studies into Zotero (USA, version 7.0 [24]) for deduplication. Two researchers (H.Z. and Z.X.) performed the screening process separately, and if there were discrepancies, they were referred to a third researcher for finalization. For the studies that met the criteria after the preliminary screen, full texts were collected and downloaded, and unqualified studies was excluded by reading the full text. Data extraction was performed for the studies that met the inclusion criteria, including first author, publication date, country, total sample size, gender, age, outcome measures, and quality score. The screening process is detailed in the PRISMA flowchart shown in Figure 1 [25].

### 2.4. Quality Assessment of Included Studies

To evaluate the quality of the included studies, we performed two levels of appraisal. First, we assessed the risk of bias for each study using appropriate tools. For randomized controlled trials, the Cochrane Risk of Bias 2 (RoB 2) tool was used [26]. We examined domains such as randomization process, allocation concealment, blinding, incomplete outcome data, and selective reporting, rating each domain and the overall risk of bias.

Second, recognizing that quality of implementation reporting is crucial in educational interventions, we additionally employed the Template for Intervention Description and Replication (TIDieR) checklist [27]. The TIDieR is a 12-item guideline specifically designed to ensure interventions are described with sufficient detail for replication. We used the TIDieR as a framework to judge whether each study had thoroughly reported the flipped classroom intervention components (e.g., what materials were used for preclass learning, how in-class activities were structured, instructor training, etc.). This served as an indicator of methodological quality in terms of transparency and replicability of the pedagogy. Using the TIDieR in a systematic review context is akin to assessing the “treatment fidelity” and clarity of intervention reporting. Each study was thus examined for both internal validity (risk of bias) and quality of intervention reporting.

### 2.5. Statistical Analysis

Statistical methods were performed using the Review Manager 5.3 and Stata 15.1 software for statistical processing. The trial data were continuous variables; thus, the mean difference (MD) and 95% confidence interval (CI) were used as the effect size for combining effect sizes. Before combining the effect sizes, heterogeneity testing was conducted on all data using the chi-square test: if *p* > 0.1 and I2 ≤ 50%, the heterogeneity between studies was considered low, and a fixed-effect model was chosen for meta-analysis; if *p* ≤ 0.1 or I2 > 50%, the heterogeneity between studies was considered high, and a random-effects model was selected. The combined effect size obtained by the fixed effects model is applicable only to the specific research population included in the analysis and cannot be easily extended to other populations or situations. The random effects model takes into account the heterogeneity among studies. Its combined effect size represents the average effect size of all possible studies and has wider applicability. After combining the effect sizes, if *p* < 0.05, it indicates a significant difference between the experimental and control groups, and the results of the meta-analysis are statistically significant. At the same time, subgroup analysis was used to explore the potential impact of variables on the results. Finally, we used funnel plots to observe publication bias. The more symmetrical the scatter points were, the lower the risk of publication bias was considered, and vice versa. Egger’s test was employed to detect publication bias in the included studies [28]. The statistical measure for detecting publication bias in Egger’s test is the intercept a and its *p*-value, and the presence of publication bias is judged by whether the 95% CI includes 0. If the *p*-value for intercept a is less than 0.05 or the 95% CI does not include 0, it suggests the presence of publication bias; otherwise, no publication bias is suggested. A *p*-value of less than 0.05 was considered statistically significant. Additionally, the T-value is a key statistic to determine whether a meta-analysis has publication bias, which is achieved by testing whether the intercept term deviates significantly from zero [29].

## 3. Results

### 3.1. Results of Literature Search

As shown in Figure 1, researchers identified 492 articles from database searches and manually retrieved 3 additional articles, totaling 495 articles. Subsequently, 44 duplicate articles were removed. After reviewing the abstracts of the remaining 451 articles, 325 articles were excluded. Among these, 21 were excluded because of non-RCT study designs; 77, for being conference abstracts; 211, for nontarget study populations; and 16, for nontarget outcomes. Upon full-text review of the remaining 126 articles, an additional 114 articles were excluded, 108 for nontarget outcomes, 3 for cross-sectional study designs, and 3 for significant differences among study populations. Ultimately, 12 articles were included in this study, 9 from China, 2 from Spain, and 1 from Iran, as shown in Table 1.

### 3.2. The Result of Quality Assessment of Included Studies

As shown in Supplementary Table S1, this study utilized the ROB 2.0 scale to assess the quality of the original studies. All studies were considered to have low risk of bias arising from the randomization process; three studies were considered concerning in terms of bias because of deviation from the intended intervention, with all others considered to have low risk; two studies were considered concerning in terms of bias because of missing outcome data, and one study was considered to have high risk; one study was considered concerning in terms of bias in the measurement of the outcome; and one study was considered concerning in terms of reporting on the selection of outcomes of bias as needing attention.

All studies provided information in four areas: description of the intervention, theory of the intervention, materials used for the intervention, and how the intervention was delivered. Three studies did not provide detailed information about the intervener, only vague information; all studies reported how the intervention was delivered to subjects, where it was carried out, and the duration, intensity, and content of the intervention; and six studies did not mention whether an individualized intervention was developed. Specific details can be found in Supplementary Table S2.

### 3.3. Results of Meta-Analysis

**Figure 2:**
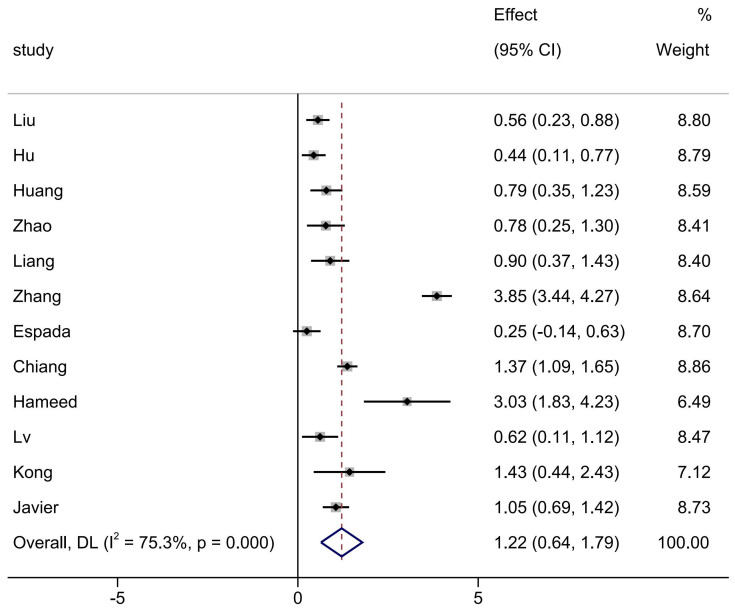
The results of the meta-analysis. The dotted line in the text is the standardised mean differences after combining the effect sizes.

As shown in Figure 2, heterogeneity testing was initially conducted, and an I^2^ value of 75.3% was found, indicating high heterogeneity. Therefore, a random-effects model was selected for analysis. The results indicated that, compared with traditional PE teaching methods, the flipped classroom model significantly outperformed traditional teaching in improving students’ motor skill scores (SMD = 1.22, 95%CI = 0.64–1.79, *p* < 0.0001), and this difference was statistically significant.

### 3.4. Subgroup Analysis

#### 3.4.1. Subgroup Analysis by Student Population

**Figure 3:**
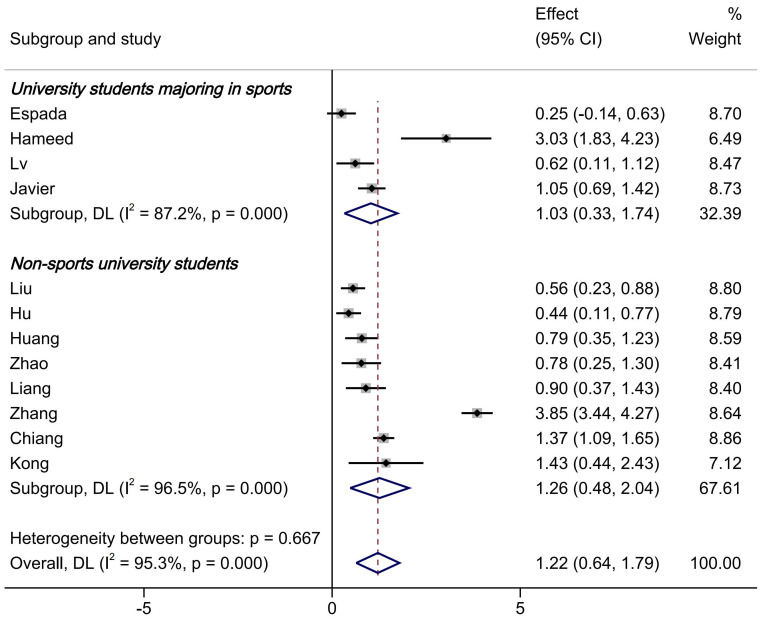
Subgroup analysis results by student population. The dotted line in the text is the standardised mean differences after combining the effect sizes.

The study then conducted a subgroup analysis based on the student population, dividing it into general major university students (for example, subjects such as biology, mathematics, and literature) and sports science major university students (in practice, these programs encompassed both applied physical education training (e.g., skill acquisition, pedagogy) and core exercise and sports science domains (e.g., physiology, biomechanics, motor control). Functionally, they were equivalent to “kinesiology” or “sport science” programs in other countries, which often confer degrees in science or health sciences). Four original studies involved sports science graduate students, while eight original studies involved general major university students. As shown in Figure 3, the flipped classroom PE model significantly outperformed traditional teaching methods in improving university students’ motor skill scores (SMD = 1.03, 95%CI = 0.33–1.74, *p* < 0.0001), and this difference was statistically significant. For general major university students, similar results were found (SMD = 1.26, 95%CI = 0.48–2.04, *p* < 0.0001). However, it is important to note that high heterogeneity remained. When the heterogeneity was analyzed, it was found that the I^2^ of sports science major students was 87.2% and that of non-sports-science-major students was 96.5%. Heterogeneity was high.

#### 3.4.2. Subgroup Analysis by Region of Study

**Figure 4:**
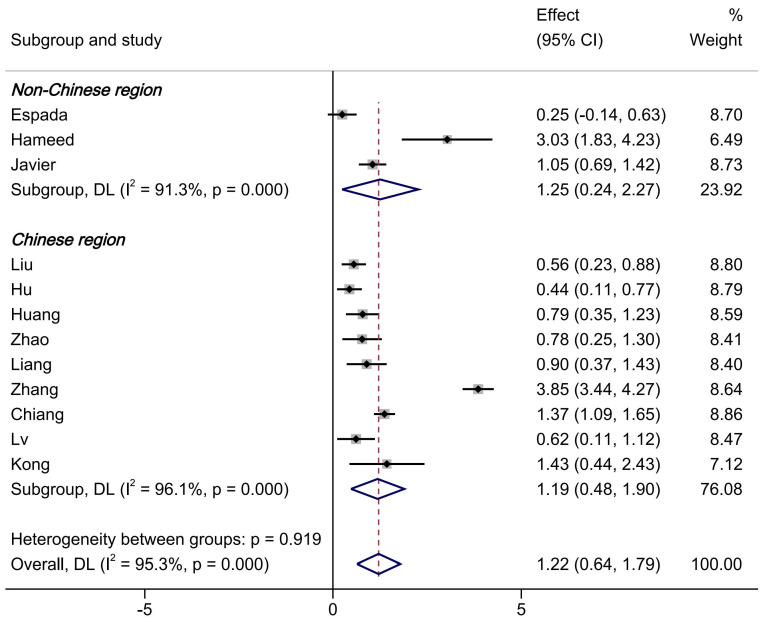
Subgroup analysis results by region of the original studies. The dotted line in the text is the standardised mean differences after combining the effect sizes.

A subgroup analysis was conducted based on the region of the original studies, dividing them into studies from China and studies from non-China regions. As shown in Figure 4, in China, the flipped classroom PE model significantly outperformed traditional teaching methods in improving university students’ motor skill scores (SMD = 1.19, 95%CI = 0.48–1.90, *p* < 0.0001), and this difference was statistically significant. Similar results were found in studies from non-China regions (SMD = 1.25, 95%CI = 0.24–2.27, *p* < 0.0001). However, it is important to note that high heterogeneity remained. When heterogeneity was analyzed, it was found that the I^2^ of the non-Chinese subgroup was 91.3%, and that of Chinese subgroup was 96.1%. Heterogeneity was high.

### 3.5. Egger’s Test

To observe whether there was publication bias among the studies, an Egger’s test was conducted. The results were as follows: standard error (SE), 1.3843; *p* value, 0.426; 95% confidence interval, [−1.566, 3.861]. This indicates that there was no significant publication bias in the aforementioned studies and that the results were robust.

**Figure 5:**
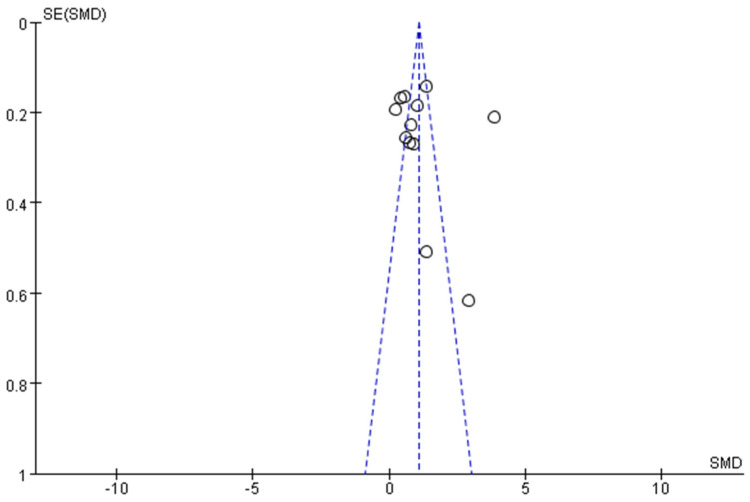
Funnel plot. The sloping dotted lines are confidence intervals, the vertical dotted lines are combined effect sizes, and the circles represent the included research studies.

The visual funnel diagram showed that the distribution on both sides of the funnel was uniform, as shown in Figure 5. According to the Egger’s test results, there was no significant publication bias.

## 4. Discussion

This study employed a meta-analysis to evaluate the effectiveness of the flipped classroom model in PE compared with that of traditional teaching methods in improving the motor skill performance of university students. The results indicated that the flipped classroom model significantly outperformed traditional teaching methods (SMD = 1.22, 95%CI = 0.64–1.79, *p* < 0.0001). This finding reveals the potential and advantages of flipped classroom as an innovative teaching model in university PE, providing empirical support for the reform of university PE.

The findings of this study are consistent with previous research, further validating the advantages of the flipped classroom in teaching effectiveness. Numerous studies have pointed out that the flipped classroom, by enhancing students’ autonomy and classroom engagement, significantly improves students’ knowledge acquisition and skill development. For instance, Hu et al. [18] found that the flipped classroom significantly improved students’ free-throw skills in basketball, and Huang et al. [32] observed that the flipped classroom significantly increased students’ movement skill scores in tai chi. These results were in line with the meta-analysis results of this study, supporting the effectiveness of the flipped classroom in improving university students’ motor skill performance. It is worth noting, however, that these results should be treated conservatively, especially when it comes to the multiple complexities of teaching content and motor skill assessment methods. Nonetheless, with the currently available evidence, they present an optimistic and positive sign.

However, some studies have failed to observe significant effects of the flipped classroom and even suggested that it may be inefficient in certain circumstances [41,42]. This may be related to the implementation conditions of the flipped classroom and individual differences among students. For example, students’ motivation to learn and self-directed learning abilities and teachers’ experience in instructional design may affect the actual effectiveness of the flipped classroom. Additionally, different sports (such as team sports versus individual sports) may have significant differences in teaching objectives and skill assessment methods, leading to heterogeneity in study results.

The teaching advantages of the flipped classroom model can be interpreted from both theoretical and practical perspectives. From a theoretical standpoint, the flipped classroom model reflects the core ideas of constructivist learning theory and self-directed learning theory [43]. Constructivism emphasizes the learner’s proactivity in knowledge construction, and the flipped classroom provides opportunities for students to explore and absorb knowledge through self-directed learning before class. In classroom practice, students can further deepen their understanding and application of motor skills through teacher–student interaction and group collaboration [44].

From a practical standpoint, the flipped classroom model has shown unique adaptability in PE. PE is practice-oriented, and the flipped classroom optimizes the efficiency of classroom time by separating theoretical learning from practical operations. In the preclass preparation phase, students can use teaching videos or other multimedia resources for prelearning; during the classroom phase, more time can be spent on skill training and teacher–student communication, enhancing the specificity of teaching. These features give the flipped classroom a significant advantage in improving motor skill performance [45].

### 4.1. Strengths of This Study

The results of this study have important guiding significance for university PE reform. In traditional teaching models, students are often passive, with low interest in learning; the flipped classroom, by adjusting teaching structures and optimizing resource allocation, effectively stimulates students’ motivation to learn, laying the foundation for developing a lifelong awareness and ability in sports [46].

From a psychological perspective, the following reasons may explain why the flipped classroom is more likely to improve students’ athletic performance. The flipped classroom takes students as the main body, emphasizing that students master theoretical knowledge through self-directed learning before class. This process aligns with previous studies [47] and with the postulates of self-determination theory [48], which posits that when students feel autonomy and control in learning, their intrinsic motivation significantly increases. This intrinsic motivation enhances students’ cognitive engagement with knowledge, leading to greater focus and positivity in practical sessions. The flipped classroom separates theoretical learning from practical training, and the self-directed learning before class allows students to understand the key points of skill movements in advance. This advanced cognitive preprocessing process is in line with working memory theory [49], which suggests that by reducing the load of theoretical information in class, students can allocate more cognitive resources to movement adjustments and skill optimization in practice. The flipped classroom emphasizes interactivity in class and immediate feedback between teachers and students. According to social learning theory, students are more likely to internalize complex skill movements through observation, imitation, and practice [50].

Another key consideration is the integration of cognitive and neural aspects with physical skill learning—a focus of this special issue on exercise, cognition, and brain health. Cognitive–motor integration refers to the concurrent engagement of cognitive processes and motor actions, requiring the coordination of large-scale brain networks [51]. In traditional instructional approaches, physical education classes often emphasize repetitive physical practice, with relatively less cognitive engagement in decision-making or problem-solving. In contrast, a flipped classroom might naturally increase cognitive involvement by challenging students to apply prelearned knowledge during physical activities. For instance, students might need to recall rules or strategies learned beforehand and make split-second decisions in gameplay or skill execution tasks—effectively integrating thought and action. Research in motor learning supports the idea that engaging the brain during physical practice can enhance skill acquisition: practice conditions that require greater cognitive effort (e.g., varied or “interleaved” practice) lead to better long-term motor learning [52]. This suggests that pedagogical strategies prompting students to think critically while moving could strengthen the neural pathways underlying motor skills. We use the term “brain-mediated motor skill performance” to highlight outcomes where improvements in motor skills are accompanied by, or dependent on, cognitive processes in the brain. University students, whose cognitive functions are in a mature stage, present an ideal population to benefit from such an enriched learning approach that merges cognitive challenge with physical practice.

### 4.2. Limitations of This Study

However, at the same time, in order to avoid exaggerating the results of this study and causing misunderstandings among readers, it is necessary to admit the corresponding limitations of this study. There was a certain degree of heterogeneity in the meta-analysis (I^2^ = 75.3%), indicating significant differences in results between studies. Subgroup analysis showed that the flipped classroom demonstrated significant teaching effects in both general major university students and sports science major students. However, this heterogeneity may stem from several aspects. Studies in China tend to focus on teaching specific skills (such as basketball free-throws or tai chi movements), while studies outside of China focus more on comprehensive skill assessments, which may lead to heterogeneity. Additionally, educational culture and the distribution of teaching resources in different regions may also affect the results [53,54]. Furthermore, the type of sport may affect the adaptability of teaching models. For example, team sports (such as basketball) require more collaborative practice, while individual sports (such as yoga and tai chi) focus more on the precision and fluidity of movements [55], and these differences may lead to different manifestations of teaching effectiveness.

### 4.3. Suggestions for Future Research

To sum up, based on our research results and the related limitations, future research should be able to expand on these results from the following aspects. First, it is important to note that there was a regional bias due to the fact that most of the studies included in this study were from China, which may limit the external validity of the results. Future studies should include more high-quality RCTs from different regions to further verify the universality of the flipped classroom, and future research should consider conducting sensitivity analyses or meta-regressions to explore potential moderators and to better understand the variability in effect sizes. Second, because of the openness of PE teaching, it was difficult to implement complete blinding in the included studies, which may have led to some observer bias. Again, more studies should be included from different countries and regions, especially those outside of China, to enhance the external validity and universality of the meta-analysis results. Last, the effectiveness of the flipped classroom should be further explored in different sports and course types and in conjunction with the characteristics of student groups to develop differentiated teaching strategies. Future studies may focus on the long-term impact of the flipped classroom model on students’ motor skills, such as postgraduation sports participation or skill retention levels.

## 5. Conclusions

Based on the above, this study indicated that the flipped classroom-based PE teaching model significantly outperformed traditional PE teaching models in improving university students’ motor skill performance. This finding may have been due to the fact that flipped classroom puts more emphasis on the subject status of students in the classroom and fully mobilizes the subjective initiative of students in learning. Therefore, it is possible to consider applying this model in the future practice of teaching PE at the university level. It is expected that when using this approach, teaching practitioners will have the flexibility to make adjustments based fully on factors such as individual learning styles, teaching preferences, the age of participants, and cognitive overload considerations.

At the same time, considering the limitations found in this study, we heartily suggest that future research take into account related directions to promote the development of the field as well as the enrichment of theories.

## Figures and Tables

**Table 1:** The characteristics of the included studies.

Authors	Region	Teaching Method	Activity	Control	Duration	Study Population	Outcome Measure	Age	n
Liu [30] 2021	China	Flipped Classroom	Yoga	Traditional Teaching	4 months	Female University Students	Yoga Specialized Scores	19.4 ± 1.2	E:77 C:76
Hu [31] 2018	China	Yu Classroom	Basketball	Traditional Teaching	4 months	University Students	Free Throw Specialized Scores	NA-2	E:71 C:73
Huang [32] 2022	China	Flipped Classroom	Tai Chi	Traditional Teaching	4 months	University Students	Movement Skill Scores	NA-2	E:42 C:42
Zhao [33] 2019	China	Flipped Classroom	Yoga	Traditional Teaching	4 months	Female University Students	Yoga Specialized Scores	NA-2	E:84 C:84
Liang [34] 2021	China	Flipped Classroom	Badminton	Traditional Teaching	4 months	University Students	Forehand High Far Ball Skill Scores	NA-2	E:30 C:30
Zhang [35] 2021	China	Flipped Classroom	Dance	Traditional Teaching	4 months	University Students	Skill Scores	NA-2	E:128 C:128
Espada [36] 2020	Spain	Flipped Classroom	NA-1	Traditional Teaching	4 months	PE Majors	Skill Scores	NA-2	E:66 C:66
Chiang [37] 2019	China	Flipped Classroom	Basketball	Traditional Teaching	4 months	University Students	Crossover Step Skill Scores	NA-2	E:122 C:119
Hameed [38] 2023	Iran	Flipped Classroom	Steeplechase	Traditional Teaching	4 months	PE Majors	Skill Scores	19.5 + 0.4	E:12 C:12
Lv [39] 2024	China	Flipped Classroom	Basketball	Traditional Teaching	4 months	PE Majors	Free Throw Skill Scores	NA-2	E:32 C:32
Kong [16] 2024	China	Flipped Classroom	Volleyball	Traditional Teaching	4 months	University Students	Forehand Dribbling Skill Scores	NA-2	E:10 C:10
Javier [40] 2018	Spain	Flipped Classroom	NA	Traditional Teaching	4 months	PE Majors	Skill Scores	NA-2	E:65 C:65

E: flipped classroom teaching group; C: traditional classroom teaching group; NA-1: although the original study did report a program to develop ‘physical literacy’, it did not report what means were used (e.g., yoga, ball games, or running, etc.); NA-2: the specific ages were not reported in the original studies, but the educational stage of the students in the study population was the undergraduate/college stage after high school education and before postgraduate education, and their ages were approximately 16–24 years.

## Supplementary Materials

The following supporting information can be downloaded at: https://www.mdpi.com/article/10.3390/brainsci15050501/s1, Table S1: The results of ROB2.0; Table S2: The results of TIDieR scale; Table S3: The raw data of the included study.
