# Supplementary material for: Impact of Flipped Classroom Instruction on Brain-Mediated Motor Skill Performance in University Students: A Systematic Review and Meta-Analysis
Source: Brain Sci. 2025 May 14;15(5):501. doi: 10.3390/brainsci15050501 (PMC12110293; doi:10.3390/brainsci15050501)
Supplement: Supplementary file 1 [file brainsci-15-00501-s001.zip › brainsci-3633909-supplementary.pdf]

Supplementary Table S1. The results of ROB2.0.

Original research	Term1	Term2	Term3	Term4	Term5
Liu 2021					
Hu 2018					
Huang 2022					
Zhao 2019					
Liang 2021					
Zhang 2021					
Espada 2020					
Chiang 2019					
Hameed 2023					
Lv 2024					
Kong 2024					
Javier 2018					

Term 1: Bias arising from the randomisation process;

Term 2: Bias attributable to deviations from intended intervention;

Term3: Bias attributable to missing outcome data;

Term 4: Bias in measurement of the outcome;

Term 5: Bias in selection of the reported result.

Green squares represent low risk of bias, yellow squares represent some concerns, and red squares represent high risk of bias.

Supplementary Table S2. The results of TIDieR scale.

Original research	1.Name	2.Why	3.a)Materials Described	3.b)Materials Available	4.Procedures	5.Provider	6.Mode of Delivery	7.Location	8.When and How Much	9.Tailoring	10.Modifi cations	11.Planned Adherence	12.Actual Adherence
Liu 2021	√	√	√	/	√	/	√	√	√	×	×	/	/
Hu 2018	√	√	√	√	√	/	√	√	√	×	×	/	/
Huang 2022	√	√	√	√	√	√	√	√	√	√	×	√	√
Zhao 2019	√	√	√	/	√	√	√	√	√	×	×	√	√
Liang 2021	√	√	√	√	√	√	√	√	√	√	×	√	√
Zhang 2021	√	√	√	√	√	√	√	√	√	×	×	√	√
Espada 2020	√	√	√	√	√	√	√	√	√	√	×	√	√
Chiang 2019	√	√	√	√	√	√	√	√	√	×	×	×	√

Hameed 2023	√	√	√	√	√	√	√	√	√	√	×	√	√
Lv 2024	√	√	√	/	√	√	√	√	√	×	×	√	×
Kong 2024	√	√	√	√	√	/	√	√	√	√	×	√	√
Javier 2018	√	√	√	√	√	√	√	√	√	√	×	√	√

Supplementary Table S3. The raw data of the included study.

study	expn	expmean	expsd	conn	conmean	consd
Liu	77	92.55	4.163	76	90.24	4.156
Hu	71	71.42	11.519	73	66.63	10.273
Huang	42	81.235	6.277	42	76.342	6.103
Zhao	30	22.328	3.449	30	19.812	3.018
Liang	30	20.67	3.22	30	17.93	2.88
Zhang	128	88.73	2.33	128	78.99	2.71
Espada	66	5.075	0.886	44	4.853	0.912
Chiang	122	20.44	1.91	119	18.07	1.52
Hameed	12	7.5	0.529	12	5.75	0.622
Lv	32	78.91	8.26	32	74.06	7.48
Kong	10	4.26	0.63	10	3.22	0.81
Javier	65	13.55	5.74	66	6.92	6.78
